# Supplementary material for: MetaRibo-Seq measures translation in microbiomes
Source: Nat Commun. 2020 Jun 29;11:3268. doi: 10.1038/s41467-020-17081-z (PMC7324362; doi:10.1038/s41467-020-17081-z)
Supplement: Supplementary file 10 — Supplementary Data 7 [file 41467_2020_17081_MOESM10_ESM.zip › File2/Confidence_High_Taxonomy/112965_out.krona.html]

Javascript must be enabled to view this page.

members
magnitude
magnitudeUnassigned
count
unassigned
taxon
rank

112965\_out

3

superkingdom
2
3

3
phylum
1239

3
186801
class

186802
order
3

186806
family
1

1730
genus
1

1
142586

SRS016335\_contig\_number\_17723
species

family
186803
2

2

SRS047014\_contig\_number\_36945SRS142890\_contig\_number\_31892
39491
species
